# Validity and Reliability of the Bahasa Melayu Version of the Migraine Disability Assessment Questionnaire

**DOI:** 10.1155/2014/435856

**Published:** 2014-07-09

**Authors:** Munvar Miya Shaik, Norul Badriah Hassan, Huay Lin Tan, Shalini Bhaskar, Siew Hua Gan

**Affiliations:** ^1^Human Genome Centre, School of Medical Sciences, Universiti Sains Malaysia, 16150 Kubang Kerian, Kelantan, Malaysia; ^2^Department of Pharmacology, School of Medical Sciences, Universiti Sains Malaysia, 16150 Kubang Kerian, Kelantan, Malaysia; ^3^Gleneagles Medical Centre, No. 1, Jalan Pangkor, 10050 Penang, Malaysia

## Abstract

*Background*. The study was designed to determine the validity and reliability of the Bahasa Melayu version (MIDAS-M) of the Migraine Disability Assessment (MIDAS) questionnaire.* Methods*. Patients having migraine for more than six months attending the Neurology Clinic, Hospital Universiti Sains Malaysia, Kubang Kerian, Kelantan, Malaysia, were recruited. Standard forward and back translation procedures were used to translate and adapt the MIDAS questionnaire to produce the Bahasa Melayu version. The translated Malay version was tested for face and content validity. Validity and reliability testing were further conducted with 100 migraine patients (1st administration) followed by a retesting session 21 days later (2nd administration).* Results*. A total of 100 patients between 15 and 60 years of age were recruited. The majority of the patients were single (66%) and students (46%). Cronbach's alpha values were 0.84 (1st administration) and 0.80 (2nd administration). The test-retest reliability for the total MIDAS score was 0.73, indicating that the MIDAS-M questionnaire is stable; for the five disability questions, the test-retest values ranged from 0.77 to 0.87.* Conclusion*. The MIDAS-M questionnaire is comparable with the original English version in terms of validity and reliability and may be used for the assessment of migraine in clinical settings.

## 1. Background

Migraine is the most common type of primary headache among patients who seek medical care [1]. The global prevalence of chronic migraine ranges from 1.4% to 2.2% [2] and tends to be higher among females (18%) compared with males (7%) [1]. According to a World Health Organization (WHO) report, 3000 migraine attacks occur every day per one million people in the population [1].

Migraine is an extremely painful recurring headache that usually affects only one side of the head. It is characterized by sharp pain and is often preceded by nausea, vomiting, and visual disturbances [3]. The intensity, duration, and frequency of the headaches and the severity of the associated symptoms may vary from patient to patient or within the same patient over a given time period [4, 5]. Migraine is ranked the 19th among the causes of years lived with disability [1].

According to the Global Burden of Disease report [6], migraine is one of the leading causes of disability worldwide and can cause significant social, economic, and personal burdens [7]. According to the WHO, disability is considered to be the impact of any disease or pathological condition on an individual's ability to work and function in various settings and roles [8–10]. Disability is commonly associated with reduced productivity, which may impose a burden on both the patient's economic productivity and the country's economic productivity [11].

Information about disability among migraine patients is very important because it can complement the patients' diagnosis and help physicians in selecting specific treatment [12, 13]. The US Headache Consortium Guidelines emphasize the importance of assessing headache-related disability in the management of migraine and the need to tailor treatment for patients by developing individualized management plans [14].

The three most frequently used headache-specific outcome measures are the Migraine Disability Assessment Score (MIDAS) questionnaire [15, 16], the Headache Impact Test (HIT) [17] and the Headache Disability Inventory (HDI) [18]. Among these questionnaires, the MIDAS questionnaire is the easiest to complete and was therefore used in this study.

## 2. MIDAS Questionnaire

The MIDAS questionnaire was first reported in 1999. It is designed to assess the impact of migraine on schoolwork, paid work, household work, and family and social or leisure activities in the past three months [15, 16]. MIDAS has become a popular and useful tool for evaluating migraine-related disability worldwide.

The self-administered MIDAS questionnaire comprises seven items and is easy to score. The first five questions assess the influence of headache on three domains of activity (work, household work, and nonwork activities) over the preceding three months with scores ranging from 0 to 92. The other two questions (A and B) were designed to provide clinically relevant information on the frequency and intensity of headache and are therefore not considered in the MIDAS score.

The MIDAS score is obtained by totaling the scores for the first five questions to produce a sum ranging from 0 to 276. Four disability grades are assigned based on the total scores: grade I (total score 0–5, indicating minimal or infrequent disability), grade II (total score 6–10, indicating mild or infrequent disability), grade III (total score 11–20, indicating moderate disability), and grade IV (total score 21 or more, indicating severe disability).

A study using the MIDAS found that MIDAS scores were substantially higher among migraine patients compared with nonmigraine patients [15]. Another study [14] suggested that incorporating the MIDAS into the US Headache Consortium Guidelines can facilitate the use of stratified care strategies that are preferred over those that follow a conventional stepwise approach. The initial treatment strategies can be adopted according to the extent of the disability as indicated by the MIDAS [14].

Previous studies have indicated that the original English version [15, 16] as well as the Italian [19], Japanese [20], Turkish [21], Taiwanese [22], and Hindi versions [23] of the MIDAS questionnaire displayed good validity and reliability. However, to date, the reliability and validity of the Bahasa Melayu or Malay version of the MIDAS (MIDAS-M) have not been investigated. Therefore, this study aimed to assess the validity and reliability of MIDAS-M for migraine sufferers.

## 3. Methods

### 3.1. Forward and Back Translation

**Figure 1:**
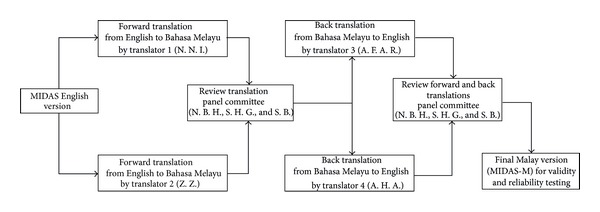
Forward and back translations of the MIDAS-M questionnaire. N. N. I.: Nik Nor Izah Nik Ibrahim, Z. Z.: Zalina Zahari, A. F. A. R.: Abdul Fatah Abdul Rahman, A. H. A.: Asma Hayati Ahmad, N. B. H.: Norul Badriah Hasan, G. S. H.: Gan Siew Hua, and S. B.: Shalini Bhaskar.

The MIDAS English version was translated into Bahasa Melayu according to standard methodology [24]. The forward translation was performed by two independent translators (N. N. I. and Z. Z.) (Figure 1). Both translators are bicultural native Malay speakers with a good command of English and speak multiple dialects of the Malay language. The forward translations were further evaluated by a panel of experts (N. B. H., S. H. G., and S. B.) to verify the semantic, idiomatic, conceptual, and cross-cultural equivalence to the English version.

The forward translation was then back-translated into English by two different independent back translators (A. F. A. R. and A. H. A.) who were unaware of the concepts and purpose of the questionnaire or the nature of the study. All of the translators were bicultural native Malay speakers with knowledge of multiple dialects and a good command of the English language. A special panel (N. H. B. and S. H. G.) reviewed the forward and the back-translated versions to produce the Malay version of the MIDAS.

### 3.2. Face and Content Validity of the MIDAS-M

To assess face validity, the MIDAS-M was administered to 30 migraine patients aged between 15 and 60 years. This step is essential to ensure the quality of the questionnaires and also to acquire proper feedback from patients while they answer the translated questions. Any difficulty in understanding the questions was noted. The content validity was established by administering the MIDAS-M questionnaire to an expert (S. B.) in the neurology department for further evaluation. This step was followed by examination by a special panel of experts (N. H. B., S. H. G., and S. B.) who reviewed, discussed, and modified the questionnaires to address the problems noted.

### 3.3. Data Collection

Registered migraine patients from the Neurology Clinic of Hospital Universiti Sains Malaysia (HUSM) were screened against the inclusion and exclusion criteria between January and May 2013. The study included patients between 15 and 60 years of age who had been diagnosed with migraine based on the criteria of the* International Classification of Headache Disorders*, 2nd edition (ICHD-II), 2004 [3]. Patients with neurological disorders, head injuries, ongoing menstrual cycle, headaches during menstrual cycle, and pregnancy were excluded. The study was approved by the Universiti Sains Malaysia Research and Ethical Committee (ethical number USMKK/PPP/JEPeM [231.3.(08)]) and complies with the Declaration of Helsinki.

After the screening process, the patients were verbally informed about the purpose of the study and completed written informed consent forms that they agreed to be enrolled in the study. Next, all of the participants were evaluated by a headache specialist during their initial visit and provided the following: (1) sociodemographic information and (2) the completed final version of the MIDAS-M questionnaire. The patients were asked to complete the MIDAS-M twice: the first time during their visit to clinic (1st administration) and the second time at home (2nd administration). For the second administration, they were requested to mail back the completed questionnaires in stamped envelopes after 21 days.

### 3.4. Statistical Analysis

An exploratory factor analysis with varimax was performed to determine the construct validity of the MIDAS-M. Kaiser-Meyer-Olkin (KMO) test and Bartlett's test of sphericity were applied to measure the sampling adequacy [25] for the factor analysis.

The internal consistency of the MIDAS-M score was assessed using Cronbach's alpha (*α*) (Cronbach's *α*). An *α* value of 0.7 is considered to be “acceptable,” while an *α* value of 0.8 or more indicates excellent internal consistency. The test-retest reliability was evaluated using Spearman and Pearson's correlations to measure the consistency over time (between the 1st and 2nd administrations) of both the total score and the individual question scores. Both correlations were utilized because the Spearman correlation tends to be conservative and is not usually influenced by outliers, whereas the Pearson correlation coefficient tends to be influenced by outliers. The statistical analysis was performed using IBM SPSS version 20.0 software (IBM Corporation, New York, USA).

A Bland-Altman plot [26] was used to confirm the agreement between the 1st and 2nd responses by calculating the mean differences between them. This method uses the mean difference between the two methods of measurement (the bias) and the 95% limits of agreement as the mean difference (1.96 SD). However, the percentage of concordance between the 2nd set of responses and the 1st was taken to represent the stability of the MIDAS scores between administrations and also the agreement between the two administrations.

## 4. Results

The Malay version of the MIDAS questionnaire was successfully administered to 30 patients with migraine to assess its face validity and was also given to experts to assess its content validity. The MIDAS-M was easily understood and well accepted by the patients.

### 4.1. Validity and Reliability of the MIDAS-M

A total of 100 patients completed the given questionnaires relatively quickly. None of the patients had any difficulties understanding or answering any parts of the MIDAS-M. The mean age of the patients was 27.95 (9.69) years, and they had a mean of 13.53 (2.67) years of education (Table 1). In this study, the majority of the patients were single (66%) and students (46%), with household incomes between RM 1500 and RM 3000 (32%). The sample included only female migraine patients, and all of the participants belonged to the Malay race, which is the dominant race in Kelantan state.

The sample was adequate according to the KMO value of 0.75 and the significant (*P* < 0.001) Bartlett's test of sphericity. On the 1st questionnaire, the mean total number of days of disability was 26.35 (25.52) days, while the mean total number of days with headache was 11.03 (10.31) days. On a scale, with a maximum value of 10, the mean pain intensity was 7.16. All patients (*n* = 100) completed the MIDAS-M questionnaires twice with no dropouts. Cronbach's *α* for the 1st administration (0.84) and the 2nd administration (0.80) exceeded the 0.8 level, indicating excellent internal consistency.

The Pearson and Spearman correlations for the 1st and 2nd administrations of the questionnaire were compared (Table 2). Except for questions A and B, the mean scores were lower on the 2nd questionnaire. There was no significant difference between the mean scores. The Spearman correlation coefficients ranged from 0.77 (for questions 3 and 5) to 0.87 for question 1 (Table 2). The total MIDAS scores for the 1st and 2nd administrations were highly correlated (Pearson's 0.91 and Spearman's 0.87) (Table 2).

For question A (total number of days with headache in the past three months), the correlation coefficients (Pearson's 0.65 and Spearman's 0.73) tended to be lower compared with the other questions. However, for question B (mean pain scale rating on the scale of 0–10), there was a strong correlation between the 1st and 2nd administrations of the questionnaire (Pearson's 0.86 and Spearman's 0.89).

**Figure 2:**
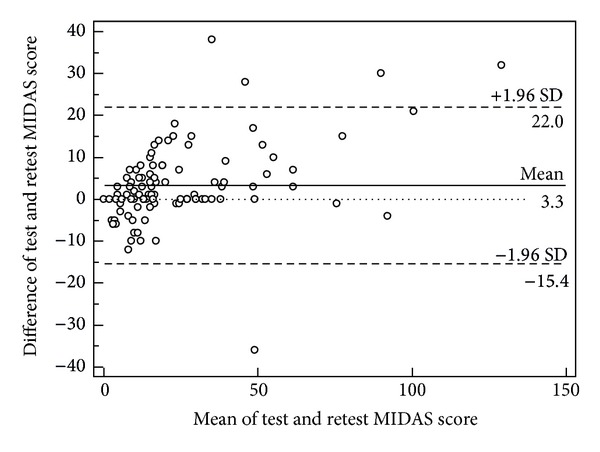
Bland-Altman plot for the repeated measurements of the MIDAS-M total scores.

Based on the Bland-Altman plot, the mean difference in the total MIDAS score between the 1st and 2nd administrations of the questionnaire was 3.3 and the 95% limits of agreement were 22 and −15.4. This result indicated the agreement between the 1st and 2nd administrations of the MIDAS-M questionnaire. However, five of the patients' total scores for the MIDAS-M questionnaires were outside the 95% limit of agreement (Figure 2).

The overall concordance was calculated as the percentage of patients who changed grades from the 1st to the 2nd administration of the questionnaire (Table 3). The overall concordance was 66% (i.e., 66 of 100 patients had the same MIDAS grade on both questionnaires). Of the patients with different grades on the 1st and 2nd administrations of the questionnaire, 79% had a difference of one grade, whereas 21% had a difference of more than one grade. The percentage of concordance was high (78%) among the patients with severe migraines but much lower (38%) among the patients who reported minimal migraine disability.

The correlation coefficients of the MIDAS-M were compared with the original and the other translated versions of the MIDAS questionnaire (Table 4). The MIDAS-M correlation values were higher compared with the original MIDAS questionnaire correlation values. The correlations for questions A and B on the MIDAS-M questionnaire were higher compared with the results of the Italian version of the MIDAS questionnaire.

## 5. Discussion

Our study is the first to assess the MIDAS questionnaire in the Bahasa Melayu or Malay language and the second assessment study conducted in Southeast Asia that follows translation and adaptation procedures. The present study indicated that the translated version of the MIDAS questionnaire was valid and reliable and has the potential to be used for quantifying migraine disability. This questionnaire is also well accepted by migraine patients. However, even though care was taken with the translation and cross-cultural adaptation process to retain the originality, simplicity, and clarity of the questionnaire, the cross-cultural equivalence of the questionnaire is subjective and difficult to measure precisely. Therefore, expert judgment was necessary to determine whether the original and translated versions were equal. A factor analysis with a good KMO value and a significant Bartlett's test of sphericity indicated the good construct validity of the questions.

During the 1st administration of the questionnaire, the patients were asked to report on any items that were unclear. None of the patients had difficulties understanding or answering the items on the MIDAS-M questionnaire. Furthermore, any ongoing therapies (symptomatic or prophylactic) that the patients were receiving were not altered during the course of the test-retest study. However, the final translated MIDAS-M was administered to 30 migraine patients for face validation to ensure the quality of the questionnaire. The patients did not have any difficulty understanding the questions, indicating that the translation was good.

There were no dropouts between the 1st and 2nd administrations since most of the patients resided around the hospital's vicinity. Apart from that, the time taken for the 2nd administration was short, overall making the patients very cooperative. The internal consistency of the MIDAS-M questionnaire was excellent in both the 1st and 2nd administrations. Cronbach's *α* value was higher than in other reported studies from USA [16], Britain [16], and Italy [19], indicating that all of the items on the MIDAS-M questionnaire measured single unidimensional latent constructs and that the questionnaire showed good reliability in measuring migraine disability among Malay patients with migraine.

The high correlation values based on Pearson's and Spearman's statistical analyses indicated the excellent test-retest reliability of the MIDAS-M questionnaire. Individual item scores did not change from the 1st administration to the 2nd administration except for item number five. This result can be attributed to the 21-day gap, which may not be ideal for assessing the number of nonworking days or the social and leisure activities foregone because of migraine. The frequency and severity of headaches were higher in the 2nd administration than in the 1st administration. This shift could also be due to the gap between the test and retest.

The total score and individual question scores were satisfactorily reproduced on both administrations. The correlation coefficients for the MIDAS-M questionnaire (both Pearson's and Spearman's) were slightly higher than the correlation coefficients for the English version of the MIDAS questionnaire [16]. However, the test-retest reliability of the two unscored questions (A and B) was satisfactory and higher than the reliability reported in other studies from USA [16], Britain [16], and Italy [19].

Question 5 produced the lowest correlation coefficient (both Pearson's and Spearman's) among the Malay patients, but the coefficients were higher than those reported for the original English version and the Italian MIDAS questionnaire [16, 19]. The discrepancies may be attributable to the different activities (social, family, and leisure) involved, which vary over time. Social and family activities are the most common activities among Malaysians, and, in the Kelantanese culture, most patients tend to engage in leisure activities on weekends. However, it is plausible that, in the three-week gap between the 1st and 2nd administrations, events may have occurred that changed the scoring for question 5.

Based on the Bland-Altman plot, the mean difference in the total MIDAS-M scores between the test and the retest was 3.3, indicating strong agreement between both administrations of the MIDAS-M questionnaire. However, this value may not cause changes in the grading unless the patients' scores were borderline to the adjacent grade. Most of the test and retest scores lay within the 95% limits of agreement, which also suggested complete agreement between the results. Only five scores lay outside the 95% limits of agreement. Most of the test and retest findings were clustered close to the mean difference line, indicating similar trends of agreement between both administrations.

In this study, the majority of patients experienced moderate to severe disability because of migraines, which may contribute to the higher grades compared with the original MIDAS study [15]. Therefore, it is plausible that patients with severe disability tend to seek advice from clinicians, whereas patients with minimal disability tend to neglect their headaches, delay a hospital visit, and rely on nonspecific migraine drugs.

Nearly 34% of the patients changed their disability grade from the 1st administration to the 2nd administration, which may be attributed to the unsuitable 21-day gap between the administrations. Additionally, the severity and frequency of headaches may have also changed during the long 21-day period before the retest, which may have resulted in alterations of the MIDAS-M grade. However, no evidence was available to help us select an appropriate time interval between the administrations of the questionnaires. Nevertheless, a gap between two days and two weeks has previously been reported as the ideal interval for test and retest reliability studies [27]. The interval in the present study was similar to that used in the original MIDAS validation and other versions of the MIDAS. It is plausible that the ideal gap may vary from one population to another. However, this hypothesis needs further investigation.

The MIDAS grade is especially useful in pharmacotherapy to encourage the rational use of drugs, especially with chronic diseases such as migraine. From the findings of our study, it is recommended that patients with minimal migraine disability be advised to take nonspecific painkillers and that those with moderate and/or severe migraine be advised to try specific migraine pharmacotherapy.

## 6. Limitations of the Study

This sample was limited only to female Malay patients because no male patients or patients of other races (Chinese, Indian, and Siamese) were registered at the clinic during the study period. Nevertheless, the possibility of menstrual migraine was excluded from the sample to avoid variations between the two administrations. The long interval between the test and retest reliability studies may have changed the migraine disability grades of the patients. Further validation of MIDAS questionnaire in a patient sample that includes both genders and all Malaysian races (Chinese, Indians, and Malays) is needed.

## 7. Conclusion

In conclusion, the MIDAS-M questionnaire maintains the brevity and simplicity of the English version and yielded slightly higher validity and reliability parameters than the original version. The MIDAS-M is helpful as a self-assessment tool for screening and grading individuals with migraine.

## Clinical Implications

Consider the following

- validation of the MIDAS questionnaire performed for the first time in Malaysia;
- forward and back translation of the MIDAS questionnaire to Bahasa Melayu;
- validation and reliability of the Bahasa Melayu version of the MIDAS questionnaire;
- MIDAS disability grading among migraine patients;
- self-assessment tool for screening and grading individuals with migraine in Malaysia.

## Figures and Tables

**Table 1:** Demographic characteristics of the subjects in the study.

	*n* (100)	Percentage (%)	Mean	SD
Age (years)			27.95	9.69
Education (years)			13.53	2.67
Relationship				
Single	66	66		
Married	31	31		
Divorced/widowed	3	3		
Employment position				
Student	46	46		
Homemaker	14	14		
Government job	27	27		
Private job	6	6		
Self-employed	5	5		
None	2	2		
Income (MYR)				
Below 450	5	5		
450–800	8	8		
801–1500	28	28		
1501–3000	32	32		
3001–6000	20	20		
Above 6000	7	7		
MIDAS grade				
Grade I	13	13		
Grade II	14	14		
Grade III	27	27		
Grade IV	46	46		

SD: standard deviation.

**Table 2:** Comparison between the total MIDAS-M scores on the 1st and 2nd administrations.

Item	Mean (SD)		Median		Pearson's	Spearman's
	1st administration	2nd administration	1st administration	2nd administration		
1	2.91 (4.95)	2.49 (3.98)	1	1	0.92	0.87
2	5.14 (6.85)	5.07 (6.09)	3	3	0.94	0.84
3	6.29 (7.23)	5.34 (6.53)	4	3	0.88	0.77
4	6.78 (6.99)	6.72 (6.15)	5	5	0.83	0.81
5	5.25 (6.37)	3.23 (4.09)	3	2	0.71	0.77

Total	26.35 (25.52)	22.74 (20.34)	18.5	15	0.91	0.87

QA	11.03 (10.31)	13.36 (12.94)	8	10	0.65	0.73
QB	7.16 (1.86)	7.44 (1.43)	7	7	0.86	0.89

**Table 3:** Changes in the MIDAS-M disability grade from the 1st administration to the 2nd administration.

MIDAS grade	Number and percentage of patients with each grade at the 1st administration	Number and percentage of patients with each grade at the 2nd administration				Number and percentage of patients who changed grades between administrations
		I	II	III	IV	
I	13 (13%)	5 (38.5%)	6 (46.2%)	2 (15.4%)	0	8 (23.5%)
II	14 (14%)	3 (21.4%)	6 (42.9%)	5 (35.7%)	0	8 (23.5%)
III	27 (27%)	1 (3.7%)	6 (22.2%)	19 (70.1%)	1 (3.7%)	8 (23.5%)
IV	46 (46%)	0	4 (8.7%)	6 (13.0%)	36 (78.3%)	10 (29.4%)

Total	100	9 (9%)	22 (22.0%)	32 (32.0%)	37 (37.0%)	34

**Table 4:** Correlation coefficients in the Malaysian, Italian, American, and English studies.

Item	Malaysia^1^		Italy^2^		USA^3^		UK^4^	
	Pearson's correlation	Spearman's correlation	Pearson's correlation	Spearman's correlation	Pearson's correlation	Spearman's correlation	Pearson's correlation	Spearman's correlation
1	0.92	0.87	0.64	0.74	0.68	0.69	0.66	0.56
2	0.94	0.84	0.77	0.81	0.54	0.65	0.71	0.65
3	0.88	0.77	0.65	0.63	0.60	0.63	0.82	0.58
4	0.83	0.81	0.72	0.64	0.64	0.59	0.60	0.56
5	0.71	0.77	0.54	0.49	0.62	0.71	0.52	0.46

Total	0.91	0.87	0.81	0.77	0.80	0.78	0.83	0.77

A	0.65	0.73	0.61	0.70	NR∗	NR∗	NR∗	NR∗
B	0.86	0.89	0.64	0.65	NR∗	NR∗	NR∗	NR∗

*NR: not reported; ^1^one hundred migraine patients from clinical studies; ^2^eighty-six migraine without aura patients from clinical series; ^3^ninety-seven migraine patients from the general population; ^4^one hundred migraine patients from the general population.

## Abbreviations

KMO: Kaiser-Meyer-Olkin
MIDAS: Migraine Disability Assessment
MIDAS-M: Migraine Disability Assessment Malay version
WHO: World Health Organization.
